# Data on phase and chemical compositions of black sands from “El Ostional” beach situated in Mompiche, Ecuador

**DOI:** 10.1016/j.dib.2020.106214

**Published:** 2020-08-21

**Authors:** Karina J. Lagos, Bojan A. Marinkovic, Anja Dosen, Marco V. Guamán, Víctor H. Guerrero, Emilio Pardo, Patricia I. Pontón

**Affiliations:** a Department of Materials, Escuela Politécnica Nacional, Quito 170109, Ecuador; b Molecular Science Institute, Coordination Chemistry Group, University of Valencia, Paterna 46980, Spain; c Department of Chemical and Materials Engineering, Pontifical Catholic University of Rio de Janeiro, Rio de Janeiro 38097, Brazil; d Department of Mechanical Engineering, Escuela Politécnica Nacional, Quito 170109, Ecuador

**Keywords:** Ferrotitaniferous sands, Ilmenite-hematite solid solution, XRF, XRPD, SEM

## Abstract

Data revealing the phase and chemical compositions of natural black sands from “El Ostional” beach, located in the northern Ecuadorian Pacific coast have been presented. The samples were collected from six points over the shore area of approximately 500 × 40 m^2^. The data on crystalline phases (iron titanium oxide, orthoclase feldspar and zircon) were determined by X-ray powder diffraction (XRPD), while semi-quantitative chemical analyses of major (Fe and Ti) and trace elements were obtained by X-ray fluorescence spectroscopy (XRF). The phase composition was verified by scanning electron microscopy (SEM), using backscattered electron (BSE) mode and energy dispersive spectroscopy (EDS). These comprehensive data are a contribution to valorize ilmenite-hematite solid solutions from natural resources towards the identification of novel technological applications.

## Specifications Table

**Table:** 

Subject	Materials Science
Specific subject area	Materials Chemistry
Type of data	Tables and Figures
How data were acquired	• Sample collection points: global positioning system (GPS). • XRPD: Bruker D8 Endeavor X-ray diffractometer with EVA and TOPAS software; PDF2 ICDD database. • XRF: Bruker S8 Tiger WDXRF spectrometer with semi-quantitative program Quant Express of SpectraPlus software. • SEM: Tescan Vega 3 and Hitachi TM3000 scanning electron microscopes.
Data format	Raw and analyzed
Parameters for data collection	• GPS: Degrees, Minutes, Seconds (DMS) coordinates. • XRPD: Cu K_α_ source, 2θ range from 10 to 80°, 0.02° step size and 2 s per step. • XRF: X-ray tube with Rh anode and 50 kV accelerating voltage. • SEM-EDS: accelerating voltage of 15 or 20 kV.
Description of data collection	• Black sand samples were collected from six points over the shore area in “El Ostional” beach. Their geographic coordinates were recorded by GPS and plotted with ArcGIS software. • XRPD: The phase composition was identified from the diffraction pattern profile refined with Le Bail method. • XRF: The chemical composition was determined by a semi-quantitative analysis. • SEM: The SEM images were obtained from BSE signal. The chemical composition was verified using EDS detector.
Data source location	The samples were collected from “El Ostional” beach, Mompiche, Ecuador. Their DMS coordinates were: (S1) 0°30′3.24″ N 80°2′19.32″ W (S2) 0°30′1.08″ N 80°2′19.68″ W (S3) 0°29′58.92″ N 80°2′20.40″ W (S4) 0°29′56.76″ N 80°2′21.12″ W (S5) 0°29′54.60″ N 80°2′22.20″ W (S6) 0°29′52.08″ N 80°2′23.64″ W The characterizations were carried out in Quito-Ecuador and Rio de Janeiro-Brazil.
Data accessibility	The data are available in this article.

## Value of the Data

• These data present the first thorough characterization of “El Ostional” beach sands from Mompiche-Ecuador, which allows comparing these black sands locally or globally.
• Dataset on phase and chemical compositions of Ecuadorian black sands could be useful as a benchmark for the identification of ilmenite-hematite solid solutions (0.6FeTiO_3_ ·  0.4Fe_2_O_3_) from natural resources.
• The data contribute to the valorization of Ecuadorian black sands as a low-cost raw material for novel technological applications of Fe_1.4_Ti_0.6_O_3_ solid solution, different from the conventional manufacturing of TiO_2_ pigment or the production of Portland cement clinker.
• In addition to spintronic devices and photocatalysts, the new applications of these black sands could include their use as a precursor for the synthesis of Fe/Ti oxide nanostructures.

## 1 Data Description

### 1.1 Data on the prospected area and sampling locations

**Fig. 1:**
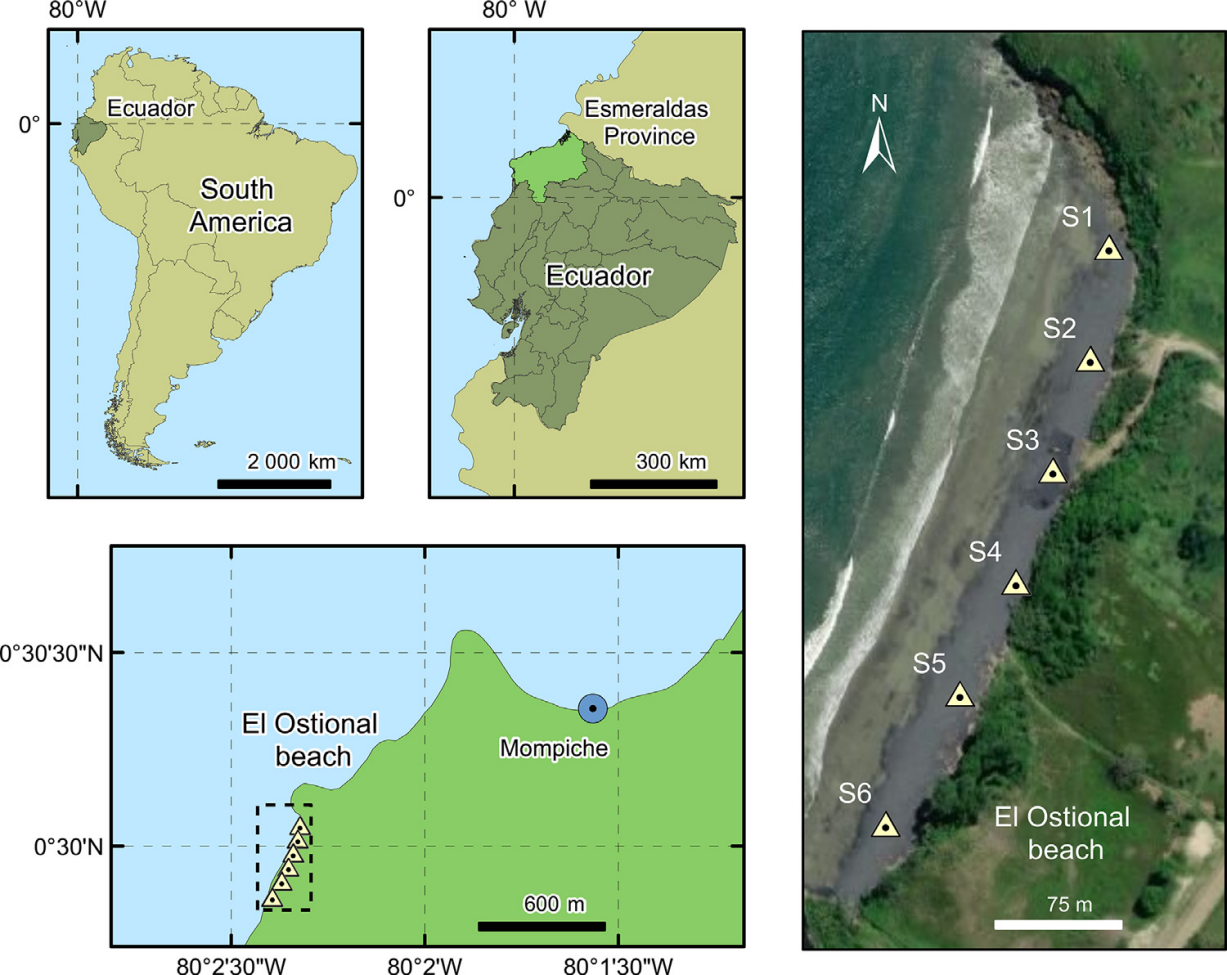
Geographic location of “El Ostional” beach and sample collection points.

Fig. 1 shows the geographic location of the prospected area that corresponds to “El Ostional” beach, situated in Mompiche-Ecuador, at the northern Ecuadorian Pacific coast. The six marks over the beach represent the collection points. The collected samples were denoted as S1 to S6.

### 1.2 Data from XRPD

**Fig. 2:**
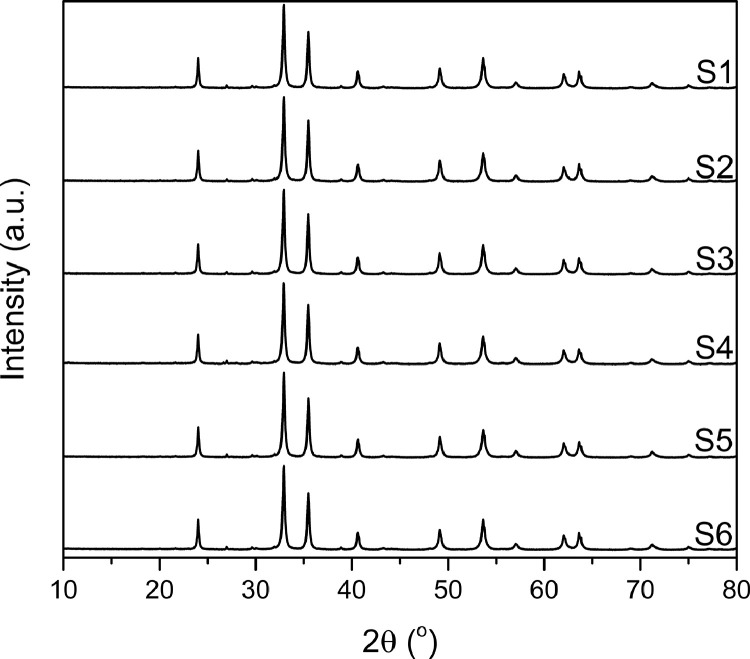
XRPD patterns of the collected samples from “El Ostional” beach.

**Fig. 3:**
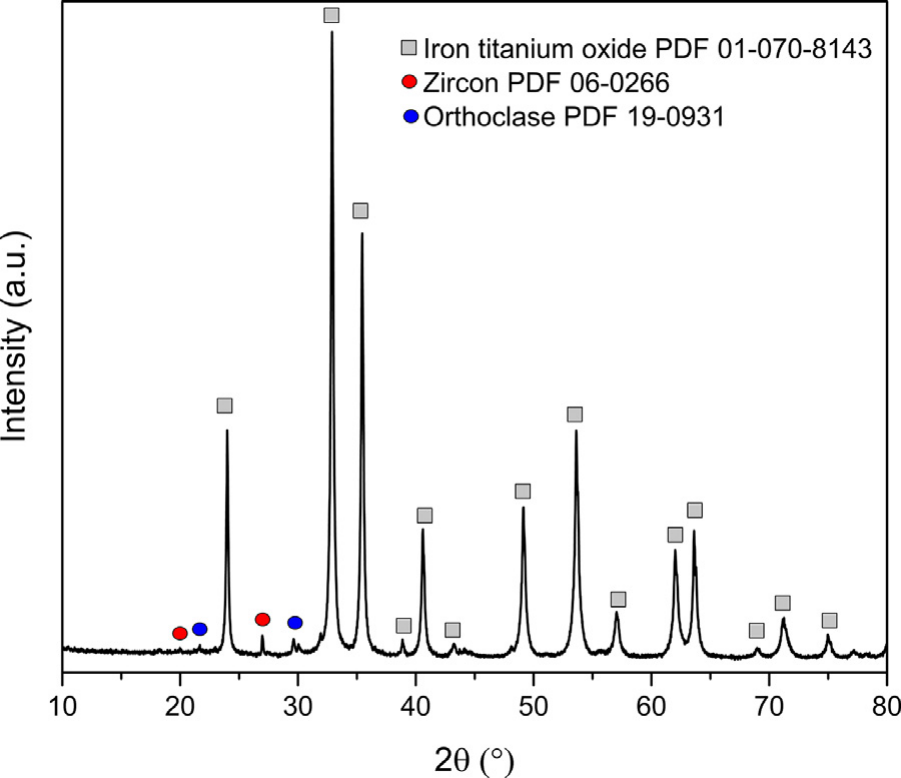
XRPD pattern of S1 sample from “El Ostional” beach with identification of crystalline phases. PDF is acronym of Powder Diffraction File database.

**Fig. 4:**
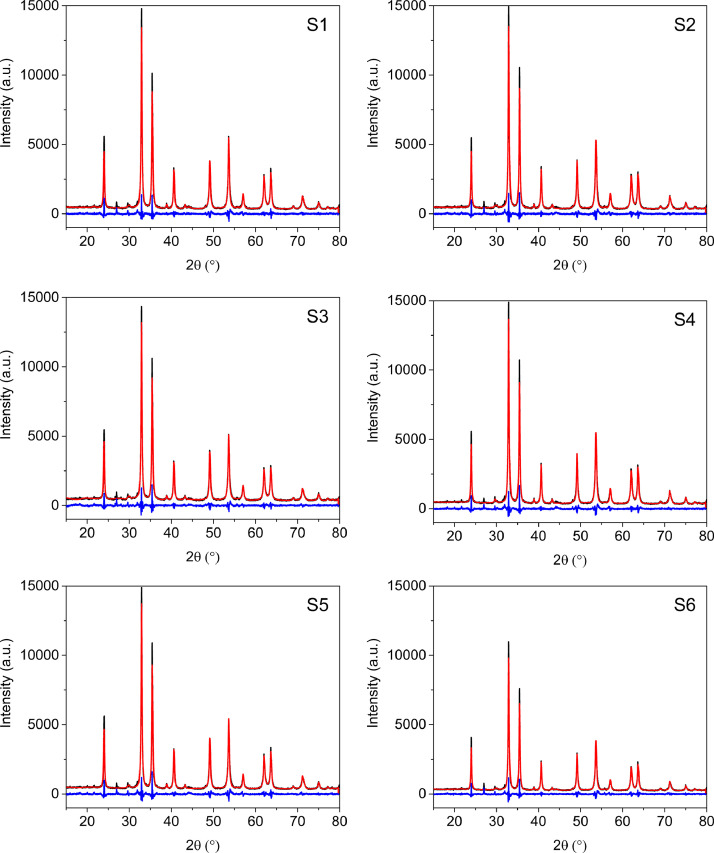
Le Bail refinements of XRPD patterns of the collected samples, S1 to S6, from “El Ostional” beach, using as the starting model for the unit-cell parameters the description of Fe_1.4_Ti_0.6_O_3_, available in PDF 01–070–8143. This is a phase within hematite-ilmenite solid solution. Experimental pattern (black), calculated pattern (red) and difference plot (blue).

Fig. 2 shows the XRPD patterns of the collected samples. All patterns exhibit the same profile (positions and intensities). Iron titanium oxide was identified as the main phase, along with trace amounts of orthoclase feldspar and zircon (Fig. 3). The phase composition was confirmed by Le Bail method [1] (Fig. 4). In the case of iron titanium oxide (Fe_1.4_Ti_0.6_O_3_), which corresponds to an ilmenite-hematite solid solution [2], Le Bail fit to the rhombohedral ordered structure within space group $R\bar{3}$ and the unit-cell parameters are presented in Table 1. In addition, TOPAS input and output data from Le Bail refinement of S1 sample are presented in Table 2.

**Table 1:** Unit-cell parameters for Fe_1.4_Ti_0.6_O_3_ samples from “El Ostional” beach.

Sample	a (Å)	c (Å)	V (Å^3^)
S1	5.061(2)	13.886(6)	308.12(3)
S2	5.061(6)	13.882(1)	307.91(9)
S3	5.062(1)	13.888(2)	308.30(12)
S4	5.061(1)	13.886(4)	308.05(19)
S5	5.061(1)	13.884(4)	308.06(21)
S6	5.062(1)	13.905(5)	308.61(22)

### 1.3 Data from XRF

Table 3 presents the semi-quantitative chemical analysis of each collected sample. Iron and titanium were identified as the major elements.

XRF data allowed verifying the chemical formula of Fe_1.4_Ti_0.6_O_3_. This solid solution belongs to Fe_(2-_*_x_*_)_Ti*_x_*O_3_ family, also represented as *x*FeTiO_3_.(1-*x*)Fe_2_O_3_ [3]. Based on the Fe/Ti mass ratio (2.8) computed from Table 3, the following equation can be written:

(1) $${\frac{Fe}{Ti}}_{\left(mass\;ratio\right)}=\frac{\left(x\right)\left(Fe_{atomic\;weight}\right)+\left(1-x\right)\left(2*Fe_{atomic\;weight}\right)}{\left(x\right)\left(Ti_{atomic\;weight}\right)}$$

where,

$$Fe_{atomic\;weight}=55.8\;\frac{g}{mol}$$

$$Ti_{atomic\;weight}=47.9\;\frac{g}{mol}$$

therefore,

$$2.8=\frac{55.8x+\left(1-x\right)\left(2*55.8\right)}{47.9\;x}$$

Thus, x=0.6

Replacing *x* value in *x*FeTiO_3_^.^(1-*x*)Fe_2_O_3_:

$$0.6\;\left(FeTiO_{3}\right)\cdot 0.4\;\left(Fe_{2}O_{3}\right)$$

$$Fe_{\left(0.6+0.8\right)}Ti_{\left(0.6\right)}O_{\left(1.8+1.2\right)}$$

**Table 2:** Input and output data from Le Bail refinement of S1 sample.

**R-Values**						
Rexp: 3.75	Rwp: 8.86	Rp: 6.76	GOF: 2.36			
**Background**						
Chebychev polynomial, Coefficient		0		408.5 (11)		
		1		−74.2 (16)		
**Instrument**						
Primary radius (mm)				280		
Secondary radius (mm)				280		
Linear PSD 2Th angular range (°)				3.958881		
FDS angle (°)				2.92327		
Beam spill, sample length (mm)				25.3068		
Intensity not corrected						
Full Axial Convolution						
Filament length (mm)				11.03792		
Sample length (mm)				11.99826		
Receiving Slit length (mm)				11.02487		
Primary Sollers (°)				2.02279		
Secondary Sollers (°)				2.025573		
Tube Tails						
Source Width (mm)				0.006448391		
Z1 (mm)				−1.191669		
Z2 (mm)				1.735961		
Fraction				0.0001724445		
**Corrections**						
Specimen displacement				−0.023 (11)		
LP Factor				0		
Surface Roughness Suortti				0.096 (8)		
Absorption (1/cm)				0 (5000)		
Sample Thickness (mm)				10 (2)		
**Miscellaneous**						
Start X				10		
**hkl Phase - 1 Le Bail method**						
Phase name				Ilmenite-hematite SS		
Space group				148		
Scale				44.3 (6)		
Cell Mass				0.000		
Cell Volume (Å^3)				307.78 (16)		
Double-Voigt|Approach						
Cry size Lorentzian				45.5 (5)		
k: 1 LVol-IB (nm)				29.0 (3)		
k: 0.89 LVol-FWHM (nm)				40.5 (4)		
Strain						
Strain G				0.00 (11)		
e0				0.0000 (2)		
Lattice parameters						
a (Å)				5.0597 (12)		
c (Å)				13.882 (3)		
				*(continued on next page)*

**Table:** **Table 2.** (*continued*)

h	k	l	m	d	2θ	I
0	0	3	2	462.740	1.916.463	0.00789
1	0	1	6	417.861	2.124.568	0.0341
0	1	2	6	370.527	2.399.773	2.72
1	0	4	6	272.059	3.289.501	13.1
1	1	0	6	252.985	3.545.423	9.28
0	1	5	6	234.528	3.834.897	3.63e-005
0	0	6	2	231.370	3.889.328	0.234
1	−2	−3	6	221.977	4.060.994	1.85
1	1	3	6	221.977	4.060.994	1.85
0	2	1	6	216.413	4.170.206	0.000252
2	0	2	6	208.930	4.326.925	0.365
0	2	4	6	185.264	4.913.747	6.9
1	0	7	6	180.674	5.047.182	0.00177
2	0	5	6	171.992	5.321.417	0.682
1	1	6	6	170.734	5.363.722	6.02
1	−2	−6	6	170.734	5.363.722	6.02
1	2	−1	6	164.451	5.586.163	0.112
2	1	1	6	164.451	5.586.163	0.112
0	1	8	6	161.337	5.703.753	2.29
1	−3	−2	6	161.095	5.713.102	0.368
1	2	2	6	161.095	5.713.102	0.368
0	0	9	2	154.247	5.991.973	0.00273
1	2	−4	6	149.470	6.204.192	4.1
2	1	4	6	149.470	6.204.192	4.1
0	2	7	6	147.029	6.318.978	0.696
0	3	0	6	146.061	6.365.741	8.75
1	−3	−5	6	142.234	6.558.111	0.0105
1	2	5	6	142.234	6.558.111	0.0105
0	3	3	6	139.287	6.715.021	0.000754
3	0	3	6	139.287	6.715.021	0.000754
2	0	8	6	136.029	6.898.154	0.772
1	0	10	6	132.339	7.119.148	3.95
1	−2	−9	6	131.698	7.159.137	0.529
1	1	9	6	131.698	7.159.137	0.529
1	2	−7	6	127.119	7.459.645	0.118
2	1	7	6	127.119	7.459.645	0.118
2	2	0	6	126.492	7.502.981	2.24
3	0	6	6	123.509	7.717.029	0.397
0	3	6	6	123.509	7.717.029	0.397
2	−4	−3	6	122.016	7.829.366	0.21
2	2	3	6	122.016	7.829.366	0.21
0	1	11	6	121.272	7.886.676	0.0343
1	−4	−1	6	121.067	7.902.669	0.0561
1	3	1	6	121.067	7.902.669	0.0561
1	−3	−8	6	119.808	8.002.329	0.596
1	2	8	6	119.808	8.002.329	0.596
1	3	−2	6	119.709	8.010.297	0.58
3	1	2	6	119.709	8.010.297	0.58

**Table 3:** Semi-quantitative chemical analysis of the collected samples from “El Ostional”.

Element	Composition (mass%)*						
	S1	S2	S3	S4	S5	S6	Average
Fe	70.13	70.64	69.13	68.08	70.48	71.00	69.91 ± 1.10
Ti	25.48	24.57	24.57	23.73	24.69	24.64	24.61 ± 0.56
Si	0.99	1.26	1.89	2.95	1.51	1.17	1.63 ± 0.72
Mg	0.65	0.75	0.79	1.33	0.55	0.63	0.78 ± 0.28
Ca	0.79	0.62	0.73	1.07	0.50	0.45	0.69 ± 0.23
Al	0.44	0.46	0.54	0.85	0.41	0.39	0.51 ± 0.17
V	0.46	0.46	0.43	0.47	0.44	0.48	0.46 ± 0.02
Zr	0.32	0.27	0.32	0.28	0.28	0.29	0.29 ± 0.02
Mn	0.25	0.25	0.25	0.28	0.24	0.23	0.25 ± 0.01
Na	0.18	0.30	0.64	0.48	0.50	0.36	0.41 ± 0.17
Cr	0.09	0.11	0.13	0.16	0.13	0.13	0.12 ± 0.02
P	0.12	0.18	0.13	0.19	0.15	0.13	0.15 ± 0.03
Zn	0.04	0.04	0.04	0.03	0.04	0.04	0.04 ± 0.00
K	0.02	0.04	0.05	0.03	0.04	0.02	0.03 ± 0.01
Pb	-	-	0.27	0.03	-	-	0.15 ± 0.17
S	0.05	0.05	0.11	0.05	0.06	0.05	0.06 ± 0.02

⁎ These data exclude the mass content of oxygen.

### 1.4 Data from SEM

**Fig. 5:**
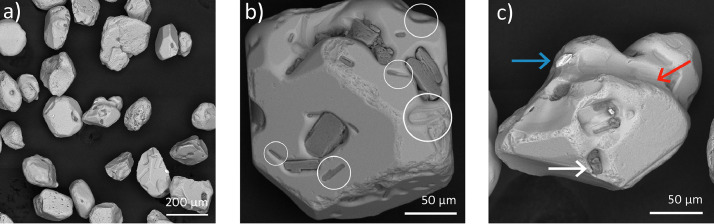
BSE-SEM images of S1 sample from “El Ostional” beach, showing (a) the morphology and size, (b) pullout of the embedded secondary particles and (c) three different mineral phases.

Fig. 5 shows BSE-SEM images of S1 sample. The particle size is between 50 and 200 µm (Fig. 5a). Irregular morphology appears to be due to weathering, which sometimes caused pullout of the embedded secondary particles (Fig. 5b). Besides, BSE signal distinguished three mineral phases which can be seen in Fig. 5c.

**Fig. 6:**
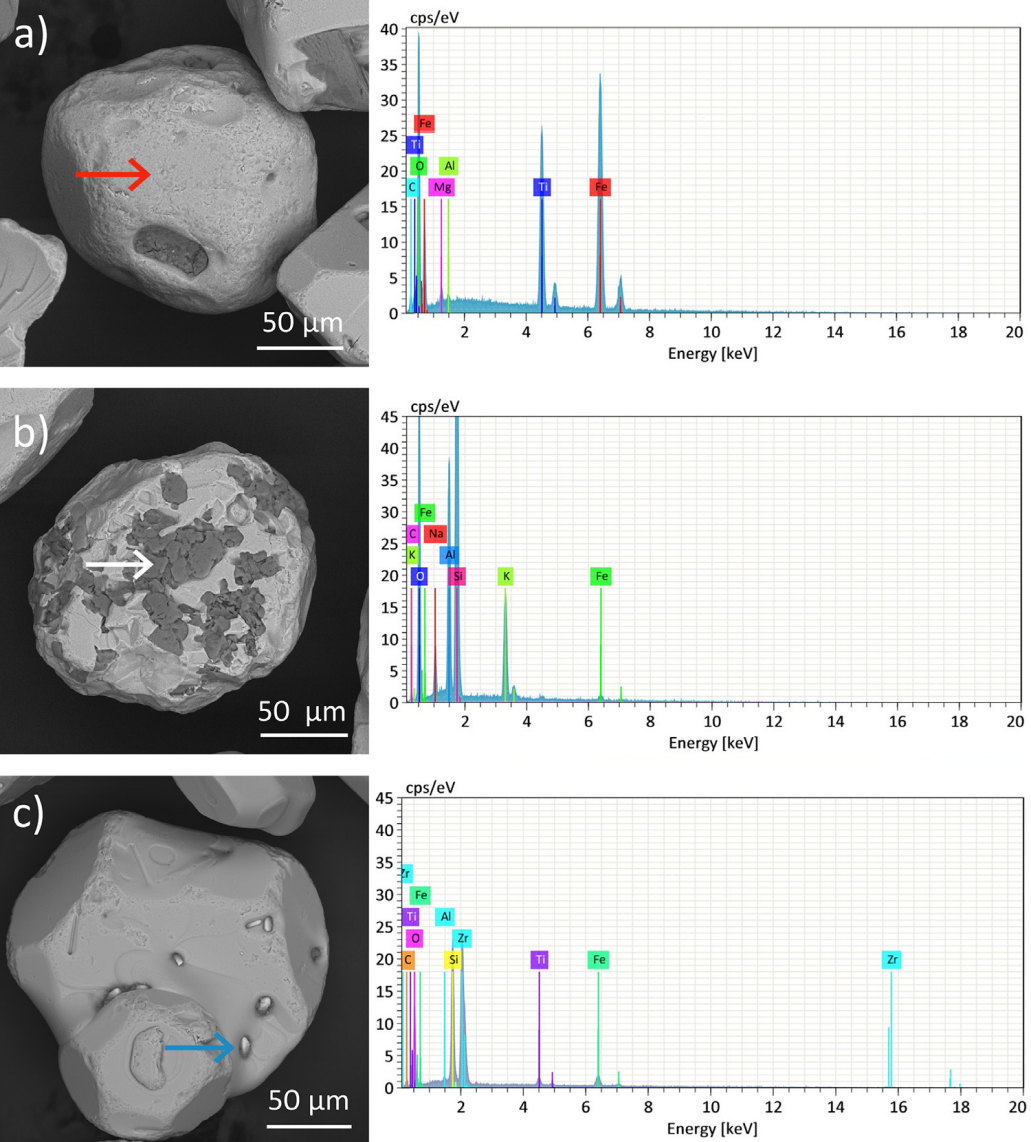
BSE-SEM images and EDS spectra of identified mineral phases in S6 sample: (a) iron titanium oxide, (b) orthoclase feldespar and (c) zircon.

The chemical composition of the identified phases was confirmed by EDS analyses. The majority phase, observed through large gray particles, is composed of Fe, Ti and O, corresponding to iron titanium oxide (Fig. 6a). Small amounts of Mg and Al were also detected. Regarding to the dark color phase (Fig. 6b), it comprises K, Al, Si, Na and O. Therefore, this signal originates from orthoclase feldspar particles. Furthermore, small white particles embedded into the iron titanium oxide (Fig. 6c) correspond to zircon, due to the presence of Si, Zr and O. Low Fe and Ti contents in secondary phases come from the surrounding iron titanium oxide.

**Fig. 7:**
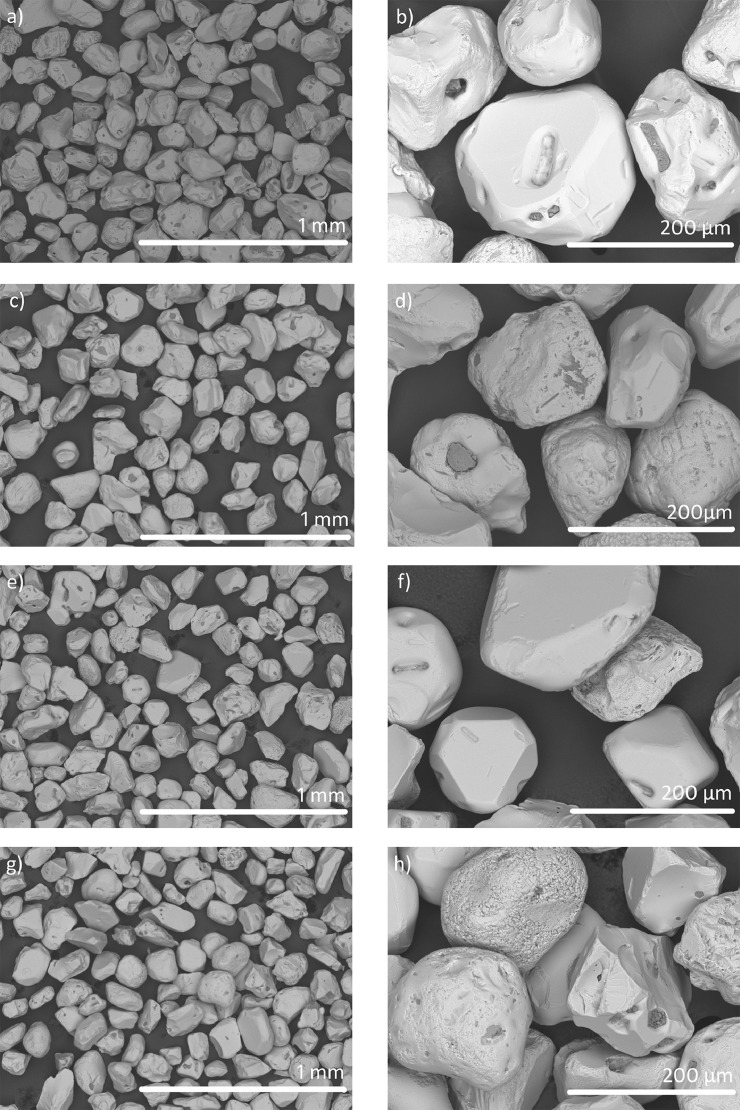
BSE-SEM images of samples from “El Ostional” beach of S2 (a, b), S3 (c, d), S4 (e, f) and S5 (g, h) samples.

All samples presented the same phases and similar particle size and morphology, as illustrated in Fig. 7.

## 2 Experimental design, materials, and methods

### 2.1 Sample collection

“El Ostional” beach (see Fig. 1) has an extension area of approximately 500 × 40 m^2^. The entire beach contains black sands over shore and offshore. The samples were merely collected from the shore. To assure a whole beach coverage, a simple systematic sampling was carried out by drawing a rectangular grid pattern comprising six 83 × 40 m^2^ cells. Thus, the black sands (5 kg) were taken from the rectangle centers at approximately 0.50 m depth. The sampling points were tracked by GPS and recorded in DMS coordinates.

Prior to analysis, all the samples were washed several times with distillated water at 50 °C to eliminate residual salt. Afterwards, they were dried in a convection oven at 80 °C for 24 h.

### 2.2 XRPD

XRPD was carried out in a D8 Endeavor X-ray diffractometer from Bruker, operating with Cu K_α_ radiation in a 2θ range from 5 to 80° and steps of 0.02°. The current of 40 mA and accelerating voltage of 40 kV (1.6 kW) were used. The phase identification was performed with EVA software and PDF2 ICDD database, while Le Bail refinement was carried out using TOPAS software. The black sands were previously ground in a RETSCH PM 400 planetary mill, with a 20:1 ball to sand mass ratio, for 3 min. The resulting powder passed through a 400 mesh screen.

### 2.3 XRF

All the samples were analyzed in the form of pressed pads, which were prepared using a sample:binder mass ratio of 9:1. A Bruker S8 TIGER WDXRF spectrometer, equipped with an X-ray tube with Rh anode, was used at 50 kV accelerating voltage. The data were analyzed by the semi-quantitative program Quant-Express of Spectra Pluss software.

### 2.4 BSE-SEM with EDS

A Vega 3 and TM3000 scanning electron microscopes from Tescan and Hitachi, respectively, were used. The raw samples were sprinkled onto a conducting tape and used as prepared, without additional carbon or gold deposition. The accelerating voltages of 15 or 20 kV were used for SEM/EDS analyses.

## Declaration of Competing Interest

The authors declare that they have no known competing financial interests or personal relationships which have, or could be perceived to have, influenced the work reported in this article.
